# Bioinspired Green Synthesis of Zinc Oxide Nanoparticles from a Native *Bacillus cereus* Strain RNT6: Characterization and Antibacterial Activity against Rice Panicle Blight Pathogens *Burkholderia glumae* and *B. gladioli*

**DOI:** 10.3390/nano11040884

**Published:** 2021-03-30

**Authors:** Temoor Ahmed, Zhifeng Wu, Hubiao Jiang, Jinyan Luo, Muhammad Noman, Muhammad Shahid, Irfan Manzoor, Khaled S. Allemailem, Faris Alrumaihi, Bin Li

**Affiliations:** 1 State Key Laboratory of Rice Biology and Ministry of Agriculture Key Laboratory of Molecular Biology of Crop Pathogens and Insects, Institute of Biotechnology, Zhejiang University, Hangzhou 310058, China; temoorahmed@zju.edu.cn (T.A.); 21916082@zju.edu.cn (Z.W.); 371112@zju.edu.cn (H.J.); nomansiddique834@gmail.com (M.N.); 2 Shanghai Extension and Service Center of Agriculture Technology, Department of Plant Quarantine, Shanghai 201103, China; toyanzi@126.com; 3 Department of Bioinformatics and Biotechnology, Government College University, Faisalabad 38000, Pakistan; mshahid@gcuf.edu.pk (M.S.); imanzoor@iu.edu (I.M.); 4 Department of Biology, Indiana University, Bloomington, IN 47405, USA; 5 Department of Medical Laboratories, College of Applied Medical Sciences, Qassim University, Buraydah 51452, Saudi Arabia; K.allemailem@qu.edu.sa (K.S.A.); f_alrumaihi@qu.edu.sa (F.A.)

**Keywords:** antibacterial activity, biosynthesis, nanopesticides, rice pathogen, ZnONPs

## Abstract

*Burkholderia glumae* and *B. gladioli* are seed-borne rice pathogens that cause bacterial panicle blight (BPB) disease, resulting in huge rice yield losses worldwide. However, the excessive use of chemical pesticides in agriculture has led to an increase in environmental toxicity. Microbe-mediated nanoparticles (NPs) have recently gained significant attention owing to their promising application in plant disease control. In the current study, we biologically synthesize zinc oxide nanoparticles (ZnONPs) from a native *Bacillus cereus* RNT6 strain, which was taxonomically identified using 16S rRNA gene analysis. The biosynthesis of ZnONPs in the reaction mixture was confirmed by using UV–Vis spectroscopy. Moreover, XRD, FTIR, SEM-EDS, and TEM analysis revealed the functional groups, crystalline nature, and spherical shape of ZnONPs with sizes ranging from 21 to 35 nm, respectively. Biogenic ZnONPs showed significant antibacterial activity at 50 µg mL^−1^ against *B. glumae* and *B. gladioli* with a 2.83 cm and 2.18 cm zone of inhibition, respectively, while cell numbers (measured by OD_600_) of the two pathogens in broth culture were reduced by 71.2% and 68.1%, respectively. The ultrastructure studies revealed the morphological damage in ZnONPs-treated *B. glumae* and *B. gladioli* cells as compared to the corresponding control. The results of this study revealed that ZnONPs could be considered as promising nanopesticides to control BPB disease in rice.

## 1. Introduction

Rice (*Oryza sativa* L.) is an important cereal crop consumed by more than half of the world’s population and is a good source of proteins, carbohydrates, vitamins, and minerals. Almost 85% of rice is cultivated in Asia, whereby China is the world’s leading producer of rice [1]. The global food security challenge in the modern world is continuously increasing due to the constant increase in the world’s population, which is expected to be 9 billion by 2050 [2]. Over the last few decades, rice crops have been threatened by different bacterial phytopathogens, and plant diseases are limiting crop yields across the world. The most important rice bacterial pathogens, *Burkholderia glumae* and *B. gladioli,* are deemed to be the leading threat to rice cultivation that cause bacterial panicle blight (BPB) disease, which poses a severe threat to rice production [3,4]. 

The rice BPB disease was first reported in Japan; however, it is currently spread in most rice-producing countries, including China, the United States, Malaysia, Sri Lanka, Thailand, South Korea, and Panama [5,6]. The BPB has severely reduced yields of infected rice fields as it causes severe damage, e.g., floret sterility, grain abortion, and milling quality reduction [7,8,9]. It is widely accepted that the rice yield losses associated with BPB disease cannot be mitigated with applications of chemical pesticides [10]. The lack of effective alternative control for BPB may be due to the increased bacterial resistance, high genetic diversity, and climatic conditions in the current global warming scenario [11]. There is an urgent need to develop unique techniques for managing the rice BPB disease caused by *B. glumae* and *B. gladioli*. 

In recent years, nanotechnology has emerged as a promising research area with a wide range of applications in the agriculture sector, especially in plant disease management [12,13,14]. Moreover, NPs are low-cost, efficient alternatives to parent materials with high reaction rates, superior efficiency, and a large surface/volume ratio [15,16]. In the past few years, NPs have been synthesized through physical and chemical methods, which had less biocompatibility, higher production rates, required the use of hazardous chemicals, and had high energy requirements [17]. In the previous literature, the use of zinc oxide nanoparticles (ZnONPs) as an antibacterial agent is a well-known effective approach and has received considerable attention in plant disease management due to their unique physico-chemical properties [18,19,20,21]. Moreover, numerous studies have reported that ZnONPs significantly increase plant growth and inhibit the growth of broad-spectrum pathogens [22,23]. An earlier study reported that green ZnONPs significantly inhibited the growth of rice pathogens *Xanthomonas oryzae* pv. *oryzae* [24]. Similarly, Rajabairavi et al. [25] revealed the antibacterial effect of microbe-mediated ZnONPs against two bacterial pathogens, *Pseudomonas aeruginosa* and *Enterobacter aerogens.* Conversely, some studies reported the adverse effects of chemically produced ZnONPs on plant soil microbial communities [26,27]. Another study performed by Zhou et al. [28] showed the adverse impact of chemically produced ZnONPs on soil microorganism biomass and rac-metalaxyl transformation. However, microbe-based synthesis of ZnONPs has enormous potential benefits in terms of long-term stability, nontoxicity, eco-friendliness, and ease of scaling-up as compared to those NPs fabricated via chemical and physical methods [22]. Nevertheless, the ameliorative effect of green ZnONPs against *B. glumae* and *B. gladioli* is not being investigated. Furthermore, a literature review is available for the potential application of biogenic ZnONPs as a nanopesticide. To the best of our knowledge, this is the first report of biogenic ZnONPs as nanopesticides against rice pathogens *B. glumae* and *B. gladioli*.

The objectives of the current study are to synthesize the biogenic ZnONPs by using a culture supernatant of native *B. cereus* strain RNT6, to evaluate their antibacterial activity against rice bacterial panicle blight disease pathogens *B. glumae* and *B. gladioli,* and to observe the antagonistic cellular interactions of ZnONPs with rice pathogens by electron microscopy analysis. 

## 2. Materials and Methods

### 2.1. Collection of Bacterial Strains 

The bacterial strain *B. cereus* RNT6 was isolated (source of biogenic ZnONPs) from soil collected from rice fields of Zhejiang University, Hangzhou, China, by using the dilution plate method [29]. In brief, 1 g of soil samples were added to test tubes containing 9 mL of sterilized saline solution (0.85% NaCl, *w*/*v*) and mixing of suspension was performed through vortex mixture (Vortex QL-861). Afterward, the 10^−4^ and 10^−6^ dilutions were spread on the nutrient agar (NA) media plates and incubated at 30 ± 2 °C for 24 h. The bacterial isolates were purified by repeated streaking for maximum purity. In this experiment, bacterial phytopathogens *B. glumae* and *B. gladioli* that cause bacterial panicle blight disease were obtained from the laboratory collection of the Institute of Biotechnology, Zhejiang University, Zhejiang, China.

### 2.2. Taxonomic Identification RNT6 Strain

The genomic DNA of *B. cereus* RNT6 was isolated using the CTAB method and quantified through Nano Drop™ 2000/2000c (Thermo-Fisher Scientific, Waltham, MA, USA). The amplification of the 16S rRNA gene was carried out with the universal primer pair (fD1 and rD1) according to the method of Weisburg et al. [30]. The amplicon was sequenced commercially from Tsingke, Beijing, China, by Sanger-sequencing technique. The 16S rRNA gene sequence of bacterial isolate RNT6 was compared with different databases for similarity search using BLASTn server at National Center for Biotechnology Information (NCBI), RDP database and EzBioCloud server. The phylogenetic tree of *B. cereus* RNT6 was formed with the MEGA 7.0 software package through the maximum likelihood method.

### 2.3. Extracellular Biosynthesis of ZnONPs

The RNT6 strain was grown in 250 mL Erlenmeyer flasks containing 100 mL of nutrient broth (NB) at 30 ± 2 °C for 24 h. After the incubation period, the bacterial culture of strain RNT6 was centrifuged at 5000× *g* for 15 min, and the collected supernatant was used for ZnONPs synthesis according to the method [31] with slight modifications. For the green synthesis of ZnONPs, 100 mL supernatant of *B. cereus* RNT6 was mixed with an equal amount of 0.1 M ZnSO_4_⋅7H_2_O in 250 mL Erlenmeyer flasks and heated on a water bath up to 80 °C for 15 min. A white precipitate starts to appear at the bottom of the flask, indicating the formation of ZnONPs. Consequently, the ZnONPs were collected by centrifuged at 15,000× *g* for 10 min and washed twice with distilled water and followed by ethanol to remove the remaining Zn^2+^ on the surface to obtain purified ZnONPs. The collected ZnONPs were then freeze-dried using Alpha 1-2 LDplus 101521 (Fisher Scientific, Waltham, MA, USA).

### 2.4. Characterization of ZnONPs

The ZnONPs were characterized by a UV-Vis spectrophotometer (Shimadzu-Kyoto, Japan) at a 250–800 nm wavelength. The size distribution of biogenic ZnONPs in water suspension with three different concentrations (10, 25, and 50 mg mL^−1^) were analyzed using a Malvern Zetasizer (Nano ZS90, Malvern, UK) according to Ahmed et al. [32]. The crystalline nature of green ZnONPs was confirmed by using XRD analysis as described by Mahdi et al. [33]. The XRD diffraction of the ZnONPs was characterized by X-ray diffractometer (Siemens-D5000, Germany). Fourier transforms infrared (FTIR) analysis was carried out according to Hossain et al. [34] to identify functional groups present in the ZnONPs. The dried sample of ZnONPs was used for the FTIR spectrum (Vector22, Bruker, Germany) in the range of 4000–500 cm^−1^. The shape, size and surface morphology of ZnONPs were observed by transmission electron microscopy (TEM) (JEM 1230-JEOL, Akishima, Japan) and scanning electron microscopy (SEM) (SU8010, Hitachi, Japan). In addition, energy dispersive spectroscopy (EDS) (Oxford Instruments, UK) was used for the conformation of elemental composition of the green ZnONPs.

### 2.5. In Vitro Antibacterial Activity of ZnONPs

The antibacterial activity of ZnONPs against rice the pathogenic bacteria *B. glumae* and *B. gladioli* was estimated through agar well diffusion according to Ibrahim et al. [35]. Briefly, 40 µL of ZnONPs with different suspensions (10, 25, and 50 μg mL^−1^) was added to a 5-mm-diameter well on Luria-Bertani (LB) agar plates; uniform swabbing was performed by bacterial culture (10^8^ CFU mL^−1^) and incubated at 30 ± 2 °C for 24 h. The diameter of the inhibition zone was calculated edge to edge across the center of disk.

Likewise, the antibacterial activity of ZnONPs against the two rice pathogenic bacteria was determined in liquid broth by measuring the optical density at 600 nm in 96-well microtiter plates (Corning, NY, USA) as per the method of Hossain et al. [36]. In general, 20 µL of pathogenic bacterial culture (10^8^ CFU mL^−1^) was added to 200 µL of LB broth containing different concentrations of ZnONPs (10, 25, and 50 μg mL^−1^) and incubated at 30 ± 2 °C for 24 h. The wells treated with 20 µL of *B. glumae* and *B. gladioli* culture without ZnONPs was used as control. Afterward, the bactericidal effect of ZnONPs on bacterial growth in the LB broth was analyzed by using a scanning microplate spectrophotometer (Thermo-Fisher Scientific, Waltham, MA, USA) at the wavelength of 600 nm.

### 2.6. Biofilm Inhibition Assay

The inhibition of *B. glumae* and *B. gladioli* biofilm by biogenic ZnONPs was measured in 96-well microtiter plates as previously described by Ogunyemi et al. [24]. The microtiter plate wells were filled with 200 mL of LB broth containing 100 mL overnight bacterial culture (10^8^ CFU mL^−1^) and ZnONPs at different suspensions (10, 25, and 50 μg mL^−1^) and incubated at 30 ± 2 °C for 24 h. Bacterial culture without ZnONPs was used as the control. Afterward, cultures were smoothly discarded and washed with sterile distilled water to remove the loosely attached cells and air-dried. Afterward, 100 μL of 1% (*w*/*v*) aqueous crystal violet solution was added for staining and kept for 30 min at room temperature. The samples intensity was found out by using a microplate spectrophotometer (Thermo-Fisher Scientific, Waltham, MA, USA) at awavelength of 570 nm after the dye was dissolved by the addition of 100 mL of 33% acetic acid.

### 2.7. Live/Dead Cell Staining

The live/dead cell staining was used to observe the membrane damage in bacterial cells by using Invitrogen, BacLight bacterial viability kit (Thermo-Fisher Scientific, Waltham, MA, USA), according to Radzig et al. [37]. Briefly, the bacterial culture (10^8^ CFU mL^−1^) was centrifuged at 8000× *g* for 5 min and the supernatant was discarded. Afterward, the bacterial cells were suspended in a 50 μg mL^−1^ concentration of ZnONPs at 30 ± 2 °C for 8 h. The live bacterial cells without ZnONPs were used as the control. Afterward, the staining reagent mixture, a red fluorescent propidium iodide (PI) stain and a green fluorescent (SYTO 9) stain, was added to the reaction mixture and incubated in the dark at room temperature for 15 min. The fluorescence emission of bacteria was assessed by means of scanning confocal laser microscopy (SCLM) (Leica-SP8, Heidelberg, Germany).

### 2.8. Flow Cytometry Observation

Flow cytometry was used to detect, identify, and count dead bacterial cells after incubation with the ZnONPs according to the method of Cai et al. [38]. The bacterial culture (10^8^ CFU mL^−1^) was centrifuged at 8000× *g* for 5 min, and pellets were treated 50 μg mL^−1^ concentration of ZnONPs at 30 ± 2 °C for 8 h. Afterward, bacterial cells were stained with red fluorescent PI stain in the dark for 20 min, andyhe cellular damage of *B. glumae* and *B. gladioli* cells was detected by using flow cytometry (Gallios-Beckman Coulter, Krefeld, Germany).

### 2.9. Determination of the Reactive Oxygen Species

ROS production in rice pathogenic bacteria *B. glumae* and *B. gladioli* was assessed by using dichlorofluorescein-diacetate (DCFH-DA) (Sigma-Aldrich, Saint Louis, MO, USA) as previously described by the method of Ogunyemi et al. [22]. In brief, 1 mL overnight culture of bacteria (10^8^ CFU mL^−1^) was centrifuged at 8000× *g* for 5 min. Afterward, the bacterial pellets were treated with 50 μg mL^−1^ concentration of ZnONPs at 30 ± 2 °C for 8 h. The nanoparticle-treated samples were gently rinsed thrice with sterilized distilled water; 10 mM DCFH-DA was added to the reaction mixture and incubated at room temperature for 10 min in a dark condition, and fluorescence was detected by using the scanning confocal laser microscopy (SCLM, Leica SP8, Germany).

### 2.10. Morphological Observation with Electron Microscopy

The morphological damage of *B. glumae* and *B. gladioli* cells in relation to the ZnONPs were observed by using SEM and TEM analysis referring to Cheng et al. [39]. Briefly, 1 mL overnight culture of bacteria (10^8^ CFU mL^−1^) was centrifuged at 8000× *g* for 5 min. Afterward, the bacterial pellets were suspended at (50 μg mL^−1^) concentration of ZnONPs at 30 ± 2 °C for 8 h. The bacterial cells without ZnONPs were used as a control. After incubation, the bacteria were washed twice with PBS and fixed in 2.5% (*v*/*v*) glutaraldehyde in 0.1 M PBS overnight. The samples were fixed with (1% *w*/*v*, osmium tetroxide) for one hour at room temperature, and then specimens were dehydrated for 15 min in a series of gradients ethanol concentration (30 to 100%). Finally, the samples of *B. glumae* and *B. gladioli* cells were observed by using TEM (JEM1230, JEOL, Akishima, Japan) and SEM (TM1000, Hitachi, Japan) analysis.

### 2.11. Statistical Analysis

All data reported in this study are the means of four replicates (n = 4 ± standard deviations). The experimental data were analyzed with Statistic (version 8.1) software, and the means were compared by least significant difference (Fisher’s LSD) at the probability level (*p* ≤ 0.05) according to Steel and Torrie [40].

## 3. Results and Discussion

### 3.1. Molecular Identification Phylogenetic Analysis of RNT6 Strain

**Figure 1:**
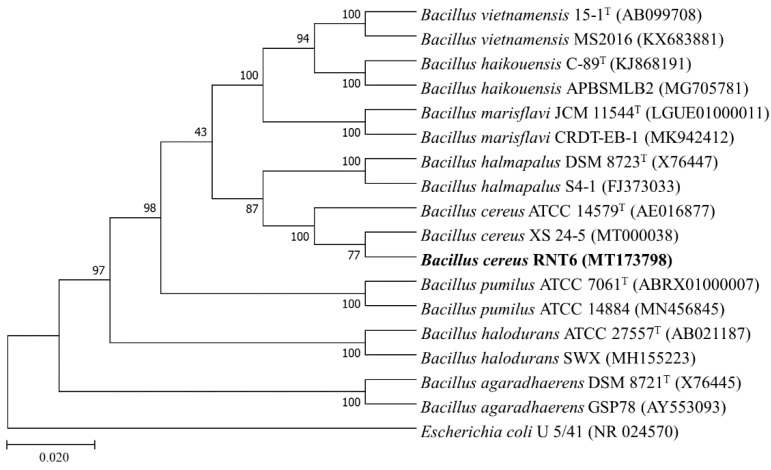
Phylogenetic analysis of *B. cereus* RNT6 with the type strains and closest Genbank matches of genus *Bacillus*.

The sequence similarity searching and phylogenetic analyses revealed the taxonomic identity of the RNT6 strain up to species level, and the name “*Bacillus cereus* RNT6 (MT173798)” was assigned to the strain. The 16S rRNA gene sequence of RNT6 strain displayed similarity with *B. cereus* XS 24-5 (MT000038; 99.86%), *B. cereus* ATCC 14579^T^ (AE016877; 99.44%), and *B. cereus* B005 (DQ289984) when searching for sequence homologues using BLASTn of NCBI, EzBioCloud, and RDP servers, respectively. The identity of the RNT6 strain was further evidenced by a phylogenetic tree, where *B. cereus* RNT6 (MT173798) shared the branch nodes with *B. cereus* XS 24-5 (MT000038), and *B. cereus* ATCC 14579^T^ (AE016877) and *Escherichia coli* U 5/41 (NR 024570) appeared as an outgroup (Figure 1). 

### 3.2. Biosynthesis and Characterization of ZnONPs

**Figure 2:**
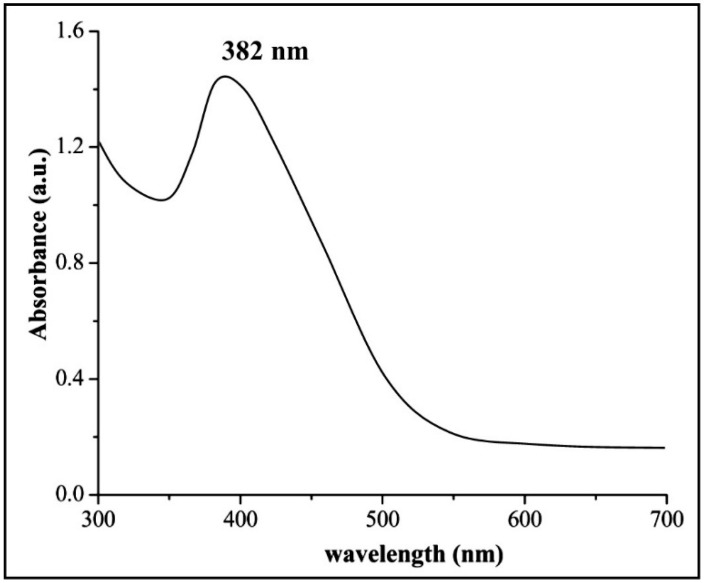
The UV–Vis spectra of reaction mixture containing stabilized biogenic zinc oxide nanoparticles (ZnONPs).

In the current study, the extracellular biosynthesis of ZnONPs was synthesized by using the *B. cereus* RNT6. The maximum precipitate clustered in the bottom of the 100 mL flask was observed at a 0.1 M ZnSO_4_⋅7H_2_O concentration. These results are in agreement with the study of Malaikozhundan et al. [41], who reported the biosynthesis of ZnONPs by using *B. thuringiensis* as a capping and stabilizing agent. Moreover, the reduction of Zn^2+^ to ZnONPs was observed through UV–Vis spectroscopy of the reaction mixture in the range of 300–700 nm. The biosynthesis of ZnONPs in reaction mixtures was confirmed through the UV-Vis characteristic peak measured at 382 nm (Figure 2). The results of UV–Vis analysis were consistent with Yusof et al. [42], who reported an absorption peak at 351 nm for ZnONPs synthesized using *Lactobacillus plantarum* TA4. The zeta potential is an important parameter that used to measure the average size and stability of NP dispersions in liquid phase. In the present study, the zeta potential and hydrodynamic diameter of three different concentrations of biogenic ZnONPs are given in Table 1. The zeta potential value of NPs > 25 mV usually has a high degree of stability [43]. The higher stability of biogenic ZnONPs has been suggested as being due to their smaller size, specific surface area and shape, and functional groups of bacteria, which did not exist in the chemically synthesized NPs. However, the biogenic ZnONPs size increased in the liquid medium as compared to the solid state, which is consistent with previously reported studies [44,45].

**Figure 3:**
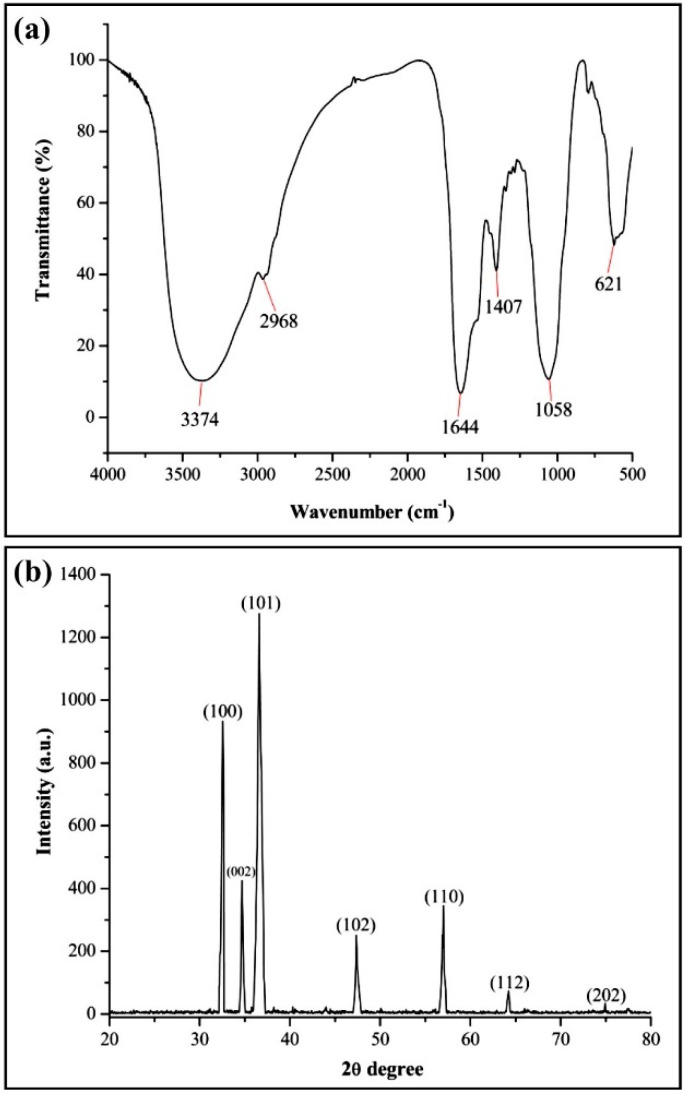
Characterization of biogenic ZnONPs synthesized from *B. cereus* RNT6. (**a**) FTIR spectra, (**b**) XRD analysis.

The FTIR analysis was used to reveal the occurrence of different functional groups that are accountable for the long-term stabilization of ZnONPs [46]. In addition, these functional groups also demonstrated the presence of macromolecules including proteins, sugars, lipids, nucleic acids, and carbohydrates that ensure the stability of biogenic ZnONPs [47]. In this study, the FTIR spectra of biogenic ZnONPs presented different absorption peaks at 3374, 2968, 1644, 1407, and 1058 cm^−1^ including a weak peak at 621 cm^−1^ (Figure 3). The peak at 3374 cm^−1^ represents the hydroxyl (O-H) group of alcohol and the peak at 2968 cm^−1^ was related to the C-H stretching of alkane group. The consistent peaks at 1644 and 1407 cm^−1^ were due to the C=N stretching of imine/oxime group and S=O stretching of sulfate group, respectively. The absorption peak at 1058 cm^−1^ revealed the presence of the S=O stretching of sulfoxide group. Our results are supported by the findings of Selvarajan and Mohanasrinivasan [31], who synthesized ZnONPs by *Lactobacillus plantarum* VITES07.

**Figure 4:**
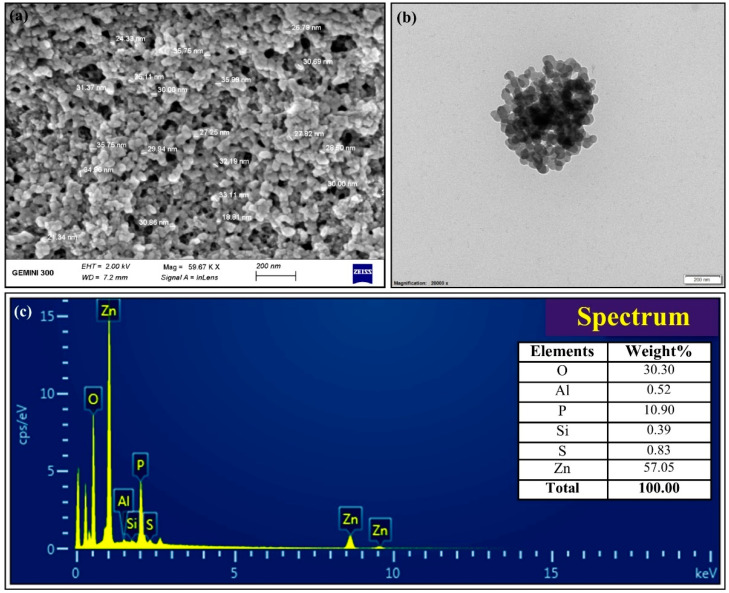
Characterization of biogenic ZnONPs through SEM-EDS and TEM analysis to observe the shape, size, and elemental composition of nanoparticles (NPs) (scale bar = 200 nm). (**a**) SEM analysis of ZnONPs, (**b**) TEM analysis of ZnONPs, and (**c**) EDS analysis of ZnONPs.

The XRD data of biogenic ZnONPs revealed the typical diffraction peaks at 32.50º, 34.74°, 36.69°, 47.33°, 56.90°, 64.32°, and 74.90°, which corresponded to (100), (002), (101), (102) (110) (112), and (202) diffraction planes of ZnONPs, respectively (Figure 3b). These XRD spectra peaks revealed the crystalline structure of the biogenic ZnONPs [48]. A similar XRD spectrum was described by Mahdi et al. [33], who produced green ZnONPs by utilizing two bacterial strains, namely, *Bacillus* sp. PTCC 1538 and *Lactococcus lactis.* Hence, the size, shape, and surface morphology of ZnONPs were observed through electron microscopy analysis [24]. The SEM and TEM analysis showed that ZnONPs have spherical shapes with the size ranging from 21 to 35 nm (Figure 4). Similarly, Mashrai et al. [49] biosynthesized spherical shape ZnONPs with a particle size of 20 nm using *Candida albicans* as eco-friendly reducing and capping agent. Furthermore, the EDS analysis revealed the presence of Zinc (57.05%), oxygen (30.30%), phosphorus (10.90%) silicon (0.39%), sulfur (0.83%), and aluminum (0.52%) in biogenic ZnONPs (Figure 4c). Our EDS results corroborate with the study of Ogunyemi et al. [22], who observed the elemental percentage, e.g., zinc (80.35%) and oxygen (19.65%) of ZnONPs. 

### 3.3. In Vitro Antibacterial Activity

**Figure 5:**
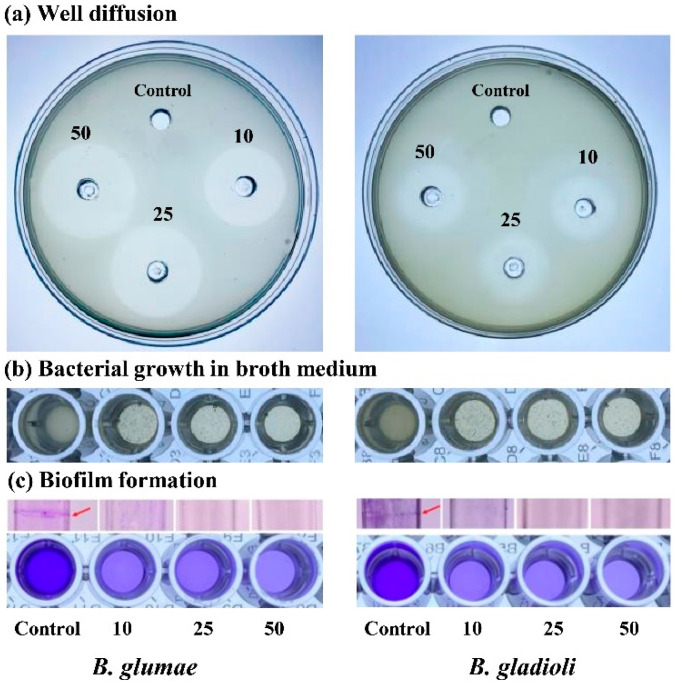
In vitro antibacterial activity of biogenic ZnONPs at three different concentrations (10, 25, and 50 µg mL^−1^) against *B. glumae* and *B. gladioli.* (**a**) The zone of inhibition was observed by well diffusion assay, (**b**) the inhibition of *B. glumae* and *B. gladioli* growth in liquid medium, (**c**) the inhibition of biofilm formation.

The growth of rice bacterial pathogens, *B. glumae* and *B. gladioli, was* inhibited significantly after the application of biogenic ZnONPs as compared to the control treatment. In the literature, various studies reported that ZnONPs effectively controlled the bacterial phytopathogens [34,50,51]. In this study, we described for the first time the antibacterial potential of biogenic ZnONPs against *B. glumae* and *B. gladioli*, which causes rice bacterial panicle blight disease. Furthermore, the zone of inhibition diameters were found to be (2.38 ± 0.15, 2.65 ± 0.05, and 2.83 ± 0.08 cm) at three different concentrations of ZnONPs (10, 25, and 50 µg mL^−1^), respectively, against *B. glumae*, whereas, for *B. gladioli*, ZnONPs revealed the diameters of zone of inhibition as 1.65 ± 0.05 cm at a 10 µg mL^−1^ concentration, 1.85 ± 0.13 cm at a 25 µg mL^−1^ concentration, and 2.18 ± 0.10 mm at a 50 µg mL^−1^ concentration. The maximum concentration, 50 µg mL^−1^, of ZnONPs demonstrated the maximum zone of inhibition diameter (Figure 5 and Table 2). Likewise, the findings of antibacterial activity in liquid broth indicated that biogenic ZnONPs significantly suppressed the growth of *B. glumae* and *B. gladioli*. The three different concentrations of ZnONPs suspension (10, 25, and 50 µg mL^−1^) caused (60.0, 66.2, and 71.2%, respectively,) reduction in the OD600 value of *B. glumae* and (52.1, 63.7, and 68.1%, respectively) reduction in the OD600 value of *B. gladioli* (Figure 5 and Table 2). Our results are constant with previous study of Jayaseelan et al. [46], who observed the growth inhibition of the bacterial pathogen *Pseudomonas aeruginosa* in solid and liquid medium by ZnONPs. Similarly, Ogunyemi et al. [22] reported the in vitro antibacterial activity (zone of inhibition and MIC at OD600) of ZnONPs against rice bacterial pathogen, while a comparatively greater inhibition effect of biogenic ZnONPs against *B. glumae* and *B. gladioli* was observed in our study as compared with the study of Ogunyemi et al. [22]. In another recent study, Yusof et al. [42] synthesized ZnONPs by using the supernatant of *Lactobacillus plantaruma* and observed their antibacterial activity against bacterial pathogens at 1000 μg mL^−1^, a high dose of ZnONPs, as compared with our study. Similarly, Venkataraju et al. [52] described the antibacterial activity of chemically produced ZnONPs (10 mg mL^−1^) against bacterial pathogens *Pseudomonas aeruginosa* and *Escherichia coli*; however, a comparatively high dose of ZnONPs was used as compared to the current study.

### 3.4. Inhibition of Biofilm Formation

Biofilm formation is a one of the major mechanisms of bacterial pathogens to adapt to various stresses and become a part of infections [53,54]. Moreover, biofilm formation most probably protects the bacterial pathogens from the immune attacks and also contribute to the survival of bacterial pathogens during the saprophytic life and latent infections [55,56]. In this study, we demonstrate that the biogenic ZnONPs significantly reduced the biofilm formation as compared to control treatment. To check the inhibition of biofilm formation, we considered three different suspensions (10, 25, and 50 µg mL^−1^) of ZnONPs. Our results revealed a 56.7, 57.6, and 65.2% reduction in the biofilm formation of *B. glumae* and a 48.9, 51.0, and 59.1% reduction of *B. gladioli* at 10, 25, and 50 µg mL^−1^ of ZnONPs, respectively (Table 2). All readings were taken at optimum OD570. Moreover, the purple biofilm circle can be clearly seen on the test tube wall, as shown in Figure 5. Our results are similar to previous studies regarding biofilm inhibition toward bacterial phytopathogens by using different nanoparticles [35,57]. Similarly, Lee et al. [58] reported the inhibition of biofilm production of *Pseudomonas aeruginosa* by using ZnONPs. 

### 3.5. Live/Dead Cell Staining 

**Figure 6:**
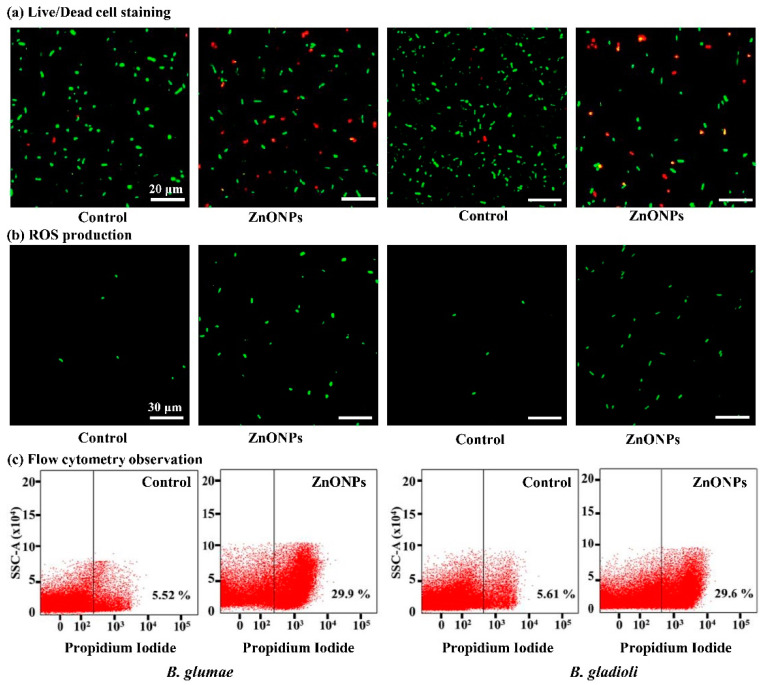
Antibacterial potential of biogenic ZnONPs against *B. glumae* and *B. gladioli* cells after 8 h treatment with (50 µg mL^−1^) and without (control) biogenic ZnONPs. (**a**) Live/dead cell staining, with green fluorescence representing the live bacteria, while red fluorescence presents dead bacteria (scale bar = 20 μm]. (**b**) Formation of reactive oxygen species (ROS) in *B. glumae* and *B. gladioli* cells. (**c**) Flow cytometry images of *B. glumae* and *B. gladioli* cells.

The results of this study demonstrated the antibacterial mechanism of ZnONPs against *B. glumae* and *B. gladioli* after application of biogenic ZnONPs. In agreement with the previous studies, ZnONPs directly changed the cellular mechanism, including cell membrane permeability, the transport of electrons, respiration, and osmoregulation, due to attachment of Zn^2+^ ions with a negatively charged bacterial cell surface that are responsible for the release of intracellular content [18,19]. Here, the staining results of the non-treated control in live bacteria evidently exhibited intact membranes that could be induced from the green fluoresce (Figure 6). Hence, the red fluorescence significantly increased after being treated with a 50 μg mL^−1^ concentration of ZnONPs at 30 ± 2 °C for 8 h compared with the control treatment. Overall, the results of live/dead cell staining revealed that the ZnONPs had a strong bactericidal effect against rice pathogens. Similarly, Cheng et al. [39] observed the red fluorescence in phytopathogen *Ralstonia solanacearum* cells after treatment by green silver NPs by using live/dead cell staining. 

### 3.6. ROS Production and Flow Cytometry Observation

ROS play an important role as cell signaling molecules for many biological processes. However, the generation of ROS can also damage cellular organelles, which can disrupt normal cellular functions [59,60]. In an earlier report, Premanathan et al. [61] reported the induction of damage in bacterial pathogens, *Pseudomonas aeruginosa* and *Escherichia coli,* due to the generation of lipid peroxidation. Inspired by previous studies, we hypothesized that ZnONPs could produce ROS that interrupted the cell membrane and infiltrated the bacterial cells, which eventually leads to the leakage of genetic material resulting in *B. glumae* and *B. gladioli* cell death. In the present study, the fluorescence intensity of *B. glumae* and *B. gladioli* significantly increased after treatment with 50 μg mL^−1^ ZnONPs as compared with the non-treated control (Figure 6). Similarly, Ogunyemi et al. [22] have found that three metal oxide nanoparticles (ZnO, MgO, and MnO_2_) can produce ROS in the rice bacterial pathogen, *Xanthomonas oryzae* pv. *oryzae*, which causes cell damage, degeneration of cell walls and cell membranes, and ultimately leads to bacterial death. Overall, our findings indicated that the antibacterial effects of ZnONPs on pathogenic bacteria *B. glumae* and *B. gladioli* are partially attributed to the production of ROS. A flow cytometry test was conducted to analyze the number of dead bacteria cells to make a quick and accurate evaluation of bacterial damage. The results of this study demonstrated that biogenic ZnONPs at a 50 μg mL^−1^ suspension significantly increased the cellular damage of *B. glumae* and *B. gladioli* (viz., 29.9 and 29.6%), respectively, while the bacterial death ratio of the negative control (without ZnONPs) was found to be 5.52 and 5.61%, respectively (Figure 6). Likewise, Abdallah, et al. [23] observed the death of bacterial cell after treatment with green chitosan and zinc oxide NPs by using flow cytometry analysis.

### 3.7. Effect of ZnONPs on Bacterial Morphology

**Figure 7:**
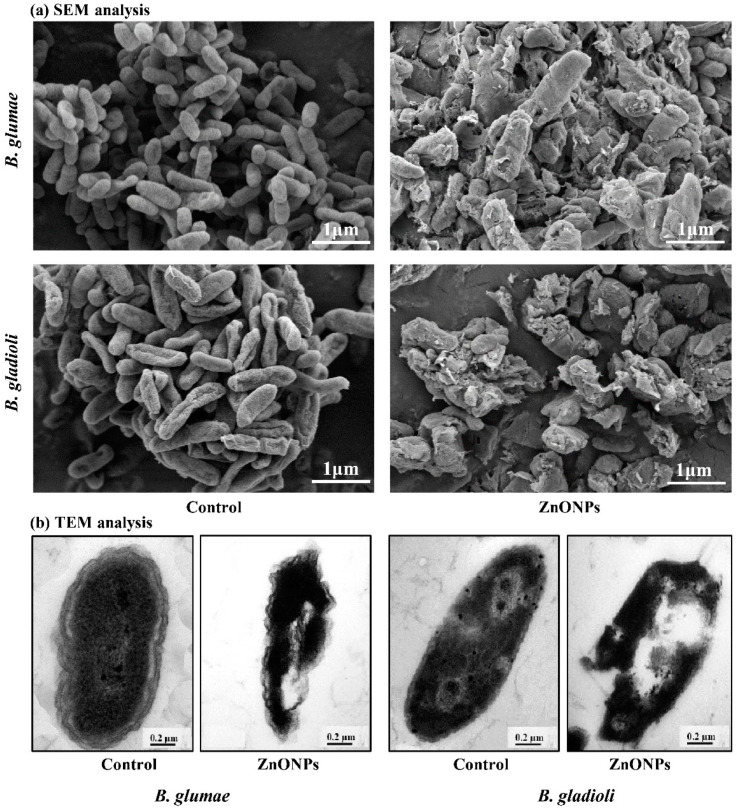
Electron microscopic SEM and TEM images of rice bacterial pathogens *B. glumae* and *B. gladioli* cells after 8 h treatment with (50 µg mL^−1^) and without (control) biogenic ZnONPs. (**a**) SEM images reveal a highly ruptured structure and small cellular holes; (**b**) TEM images show integrated bacterial membranes, disorganized cytoplasm, and leakage of bacterial genetic material.

The antibacterial potential of biogenic ZnONPs has gained tremendous attention due to their unique properties such as their ability to damage cell walls, the interrupted production of ATP, ribosomal damage, the blockage of cell transport, replication, and the leakage of genetic material [18]. Here, SEM and TEM analysis showed the ultrastructural changes in bacterial cells. After treatment with ZnONPs, the SEM micrograph showed severely disrupted and shrinkage structure of *B. glumae* and *B. gladioli* as compared with the control treatment (Figure 7). Correspondingly, TEM analysis showed that biogenic ZnONPs highly damaged cell membrane, ribosome, proteins, and cytoplasmic material that cause the leakage of nucleic acid contents, which eventually leads to the death of both pytopathogens (Figure 7). In a previous study, Ogunyemi et al. [24] observed the ruptured and collapsed cell membrane with the leakage of the cytoplasm material after treatment with plan-tmediated ZnONPs. Overall, the results of our study revealed that direct interaction between biogenic ZnONPs and *B. glumae* and *B. gladioli* caused the leakage of nucleic acid contents that results in bacterial death.

## 4. Conclusions

In the present study, we reported the potential antibacterial activity of biogenic ZnONPs against *B. glumae* and *B. gladioli* for the first time. Biogenic ZnONPs were synthesized by using *B. cereus* RNT6 strain and characterized with standard material characterization techniques. We confirmed the spherical shapes with the size ranging from 21 to 35 nm and stabilized through coating proteins in the bacterial supernatant. The biogenic ZnONPs demonstrated potential antibacterial activity against *B. glumae* and *B. gladioli*, which was revealed in terms of inhibitory zone, decrease in bacterial growth, and biofilm formation. Furthermore, ultrastructure studies revealed a wide variation in cell wall morphology and the large amount of damage to the structure of bacterial phytopathogens treated with ZnONPs (50 μg mL^−1^). Overall, the results of the current study revealed that green ZnONPs have a tendency to protect rice plants as bactericidal agents and can be utilize to produce nanopesticide formulations. However, more research insights into field conditions are needed to better understand the interactions of ZnONPs with rice plants.

## Figures and Tables

**Table 1:** Zeta potential (mV) and size of three different concentration of biogenic ZnONP suspensions in water.

ZnONPs Concentrations	Zeta Potential (mV)	Size (nm)
10 mg mL^−1^	29.5 ± 0.7	120.1 ± 6.7
25 mg mL^−1^	30.5 ± 1.1	111.9 ± 8.8
50 mg mL^−1^	31.5 ± 0.9	135.3 ± 5.3

**Table 2:** In vitro antibacterial activity of biogenic ZnONPs against rice pathogens against *B. glumae* and *B. gladioli*.

ZnONPs Concentration	Zone of Inhibition (cm)	Bacterial Growth (OD_600_)	Biofilm Formation (OD_570_)
***B. glumae***			
Control	0.00 ± 0.00	0.80 ± 0.04	1.18 ± 0.01
10 µg mL^−1^	2.38 ± 0.15 **	0.32 ± 0.02 **	0.51 ± 0.01 **
25 µg mL^−1^	2.65 ± 0.05 **	0.27 ± 0.01 **	0.50 ± 0.01 **
50 µg mL^−1^	2.83 ± 0.08 **	0.23 ± 0.01 **	0.41 ± 0.01 **
***B. gladioli***			
Control	0.00 ± 0.00	0.69 ± 0.02	0.98 ± 0.01
10 µg mL^−1^	1.65 ± 0.05 **	0.33 ± 0.04 **	0.50 ± 0.02 **
25 µg mL^−1^	1.85 ± 0.13 **	0.25 ± 0.02 **	0.48 ± 0.04 **
50 µg mL^−1^	2.18 ± 0.10 **	0.22 ± 0.01 **	0.40 ± 0.02 **

Data are presented as the mean of four replicates (n = 4 ± SE). ** Significant at *p* ≤ 0.05.

## Data Availability

The data presented in this study are available within the article.
